# ﻿A new species of the genus *Donacia* Fabricius (Coleoptera, Chrysomelidae, Donaciinae) from South Korea: morphology, ecology, and *COI* gene analysis

**DOI:** 10.3897/zookeys.1261.169034

**Published:** 2025-11-21

**Authors:** Joong Youb Kim, Yeong-Deok Han, Jong Eun Lee

**Affiliations:** 1 Research Center for Endangered Species, National Institute of Ecology, Yeongyang, Republic of Korea Research Center for Endangered Species, National Institute of Ecology Yeonyang Republic of Korea; 2 Department of Biological Science, Gyeongkuk National University, Andong, Republic of Korea Gyeongkuk National University Andong Republic of Korea

**Keywords:** Larva, new species, reed beetles, taxonomy

## Abstract

*Donacia koreana***sp. nov.** is described from South Korea, with detailed descriptions and illustrations of both the adult and larval stages. A key for species identification, distribution map, host plant associations, oviposition behaviors, feeding patterns, and other ecological information, along with partial sequences of mitochondrial cytochrome c oxidase subunit I, are also presented.

## ﻿Introduction

The genus *Donacia* Fabricius, commonly known as reed beetles or pond lily leaf beetles, belongs to the family Chrysomelidae. In BugGuide.net and iNaturalist.com, Donaciinae are referred to as aquatic leaf beetles, while *Donacia* as a genus has not been assigned a more specific common name because the genus is too diverse. It is widely distributed across the world, with many species inhabiting freshwater environments, and plays a significant role in aquatic ecosystems. Most *Donacia* species occur in diverse freshwater habitats, such as ponds, wetlands, swamps, lakes, and streams. Their unique life cycles and ecological characteristics make them important environmental indicator species (Hayashi 2020; Geiser 2023; Askevold 2024). Adults feed on leaves and flowers above the water surface, whereas larvae live underwater and feed on sap from plant roots. The larvae breathe by tapping the aerenchyma of the plant using two hollow abdominal spiracle hooks connected to their tracheal system (Geiser 2023). Askevold (in press) summarized the known research on this subject, as how donaciines obtain oxygen is not definitively demonstrated to be just one mechanism.

In South Korea, seven species belonging to the genus *Donacia* have been reported (Lee and An 2001; An 2019; Cho and An 2020; KSAE and ESK 2021; Jeong et al. 2024). Within this species list, *D. simplex* Fabricius was misidentified as *D. bicoloricornis* Chen, making their accurate differentiation crucial. In particular, Eastern Palearctic *Donacia* species are characterized by distinct differences in the presence or absence of hairs on the pronotum and elytra, which has led in part to confusion in classification (Chen 1966; Askevold 1990). Kippenberg (2015) suggested a revised arrangement of *Donacia* species, proposing several new subgenera and clarifying some existing subgeneric names. Notably, the subgenus name *Donacocia* Gistel applies to the vast majority of members of *Donaciasensu lato*. Here, the new species would be placed in the D. (Donacocia) subgenus. The two other species of *Donacia* known to be in South Korea are both in the subgenus D. (Cyphogaster) Goecke.

Adult D. (Donacocia) are typically characterized within the Donaciini tribe by the coarsely punctate-rugose pronotum, distinctly rugose to rugulose and punctulate elytra, typically entirely metallic color of elytra, pronotum and appendages. Some exception exist in which species are not metallic (e.g. *D fennica*), or with base of femora reddish (e.g. *D. bicoloricornis* and several others). Species of D. (Donacocia) are never entirely pubescent dorsally (*D. pubescens* of North America may not belong in this subgenus; Askevold in prep.)

The main morphological characteristics of *Donacia* larvae include: a slightly convex body with a very small, sclerotized, prognathous head; distinct frons, clypeus, and labrum; mandible bearing two prominent teeth; short, slightly curved, and awl-shaped legs lacking pulvilli; five stemmata on each side of the head; a thorax that is unsclerotized and covered with numerous setae; abdominal segments lacking tubercles; and a large, sclerotized spiracle hook on the 8^th^ abdominal segment (Lee 1991).

In the present study, detailed descriptions, illustrations, and taxonomic keys were developed for both adult and larval stages of the new species. Furthermore, ecological data, distribution maps, host-plant associations, oviposition behaviors, and feeding behaviors of the genus *Donacia* were synthesized, and cytochrome c oxidase subunit I (*COI*) gene sequences were analyzed.

## ﻿Materials and methods

Type specimens of the new species described have been deposited in the National Institute of Biological Resources, South Korea (**NIBR**) and J.Y. Kim’s private collection, South Korea (**JYK**).

Biological observations were conducted from May to June of 2022–2023 at the type locality and under laboratory conditions. Adults were collected by sweeping and handpicking, whereas larvae were obtained from roots of host plants. Captured adults were placed in Ziplock bags (20 cm wide, 15 cm long, and 8 cm deep) with host plants and monitored daily, whereas larvae were preserved in 70% ethyl alcohol in the field. For morphological analysis of minute structures, some larvae were dissected, cleared in 10% KOH solution for 5 h, and rinsed in distilled water. Microscopic tissues were mounted on slides, and specimens were prepared using Neo-Shigaral solution (Shiga Konchu Fukyusha, Tokyo, Japan). To examine genitalia, specimens were boiled in hot water for approximately 3 min to soften tissues. Genitalia were then carefully dissected using entomological needles and subsequently immersed in 10% KOH solution for 1–2 h to facilitate clearing. Descriptions and illustrations were prepared using a stereoscopic microscope (LEICA M125, Olympus SZX-12) and an optical microscope (Olympus BX50) equipped with a drawing tube and Adobe Photoshop CS6 software (Adobe Systems, Inc., San Jose, CA, USA) using tablets (WACOM Intuos CLT-4100). Habitus images were captured using a a Canon EOS 5D Mark 5D IV digital camera attached to a Canon EF 100 mm F2.8 Macro USM. Morphological terminology for adults largely followed Gressitt and Kimoto (1961), Kimoto and Gressitt (1979), Mann and Crowson (1983), Askevold (1987, 1988, 1990, 1991), and Hayashi (2020), whereas terminology for larvae was based primarily on Anderson (1947) and LeSage (2012).

Genomic DNA was extracted from the legs of two specimens using the LaboPas Tissue Genomic DNA Isolation Kit Mini (Cosmo Genetech Inc., Seoul, Korea) according to the manufacturer’s instructions. The *COI* barcode fragment was amplified using the universal primer set (LepF1 and HCO2198) under the following cycling conditions: initial denaturation at 94 °C for 2 min; followed by 35 cycles of 98 °C for 15 s, 53 °C for 30 s, and 68 °C for 60 s; and a final extension at 68 °C for 5 min. Amplified products were then sequenced using an ABI 3100 automated sequencer (Perkin-Elmer, CA, USA). Sequence assembly, alignment, and trimming were performed using the Geneious 8.1.9 software (Kearse et al. 2012).

*COI* sequences amplified from the newly identified *Donacia* species were aligned with those of 19 other *Donacia* species retrieved from the National Center for Biotechnology Information database, using Geneious v. 10.2.6 (Table 1). Pairwise genetic distances were calculated using the Kimura 2-parameter substitution model in MEGA X v. 12.0.11 (Kumar et al. 2024). Phylogenetic relationships were inferred using maximum likelihood analysis implemented in PhyML v. 3.1, based on the GTR + I + G model selected using jModelTest v. 2.1.10, with 1000 bootstrap replicates. The *COI* sequence of *Plateumaris braccata* (Scopoli) was used as an outgroup for this analysis.

**Table 1.** List of *Donacia* species used in molecular analysis and the respective references.

Species	Collection locality	GenBank accession No.	Reference
*Donacia koreana* sp. nov.	Korea		This study
*Donacia aquatica*	Germany	KU912172	Rulik and Ahrens 2017
	Finland	MZ659808	Roslin et al. 2022
*Donacia aureocincta*	Finland	MZ658052	Roslin et al. 2022
*Donacia bicolora*	Finland	MZ656234, MZ658244	Roslin et al. 2022
*Donacia cincticornis*	Canada	KM844344, HM411995	–
*Donacia cinerea*	Germany	KU917042	Rulik et al. 2017
	Finland	MZ658078	Roslin et al. 2022
*Donacia flemola*	China	MK049870	Nie et al. 2020
*Donacia hypoleuca*	Canada	JF887697	–
*Donacia marginata*	Germany	KU919476	Rulik et al. 2017
	Finland	KJ964703	Pentinsaari et al. 2014
*Donacia obscura*	Italy	MH323099	Magoga et al. 2018
	Finland	KJ962943	Pentinsaari et al. 2014
*Donacia piscatrix*	Canada	KM844563	–
*Donacia semicuprea*	Germany	KM443995	Rulik et al. 2017
	Sweden	KJ965000	Pentinsaari et al. 2014
*Donacia simplex*	Germany	KU913219, KU906925	Rulik et al. 2017
*Donacia sparganii*	Germany	KU910558	Rulik et al. 2017
*Donacia subtilis*	Canada	KR483642, KR486904	Hebert et al. 2016
*Donacia thalassina*	Germany	KU912621	Rulik et al. 2017
	Finland	KJ967484	Pentinsaari et al. 2014
*Donacia tomentosa*	France	KM451348	Hendrich et al. 2015
	Finland	MZ631647	Roslin et al. 2022
*Donacia versicolorea*	Germany	KU917168, KU919222	Rulik et al. 2017
*Donacia vulgaris*	Germany	KU915372	Rulik et al. 2017
	Finland	MZ631910	Roslin et al. 2022
*Donacia provostii*	Korea	OL663187	–
*Plateumaris braccata*	Finland	MZ633983	Roslin et al. 2022

## ﻿Results

### ﻿ Donacia koreana

Taxon classificationAnimaliaColeopteraChrysomelidae

Kim & Lee sp. nov.

B29FAAC6-BC1B-5DAE-888B-90C8B1C33E29

https://zoobank.org/C52E8C52-E941-4A82-B007-2242FC6BB524

Figs 1, 2, 3, 4

#### Type locality.

South Korea, Kangwon State, Hyeoncheon-ri, Dunnae-myeon, Hoengseong-gun, swamp, 37°30'9.37"N, 128°9'53.57"E.

#### Type materials.

***Holotype*** (NIBR no. NIBRIN0001069714): male, **South Korea** • Kangwon State, Hyeoncheon-ri, Dunnae-myeon, Hoengseong-gun, swamp, 37°30'9.37"N, 128°9'53.57"E, 520 m, 29.V.2022, J.Y. Kim. **Paratypes** (NIBR no. NIBRIN0001069715): • five males and seven females, same data as the holotype, except for 6.VI.2023, J.Y. Kim.

#### Other materials.

Twenty larvae were collected from the roots of *Scirpus wichurae* Boeckeler with the same data as for the paratype, except for 6.VI.2023.

#### Diagnosis, adult (Figs 1, 2, 4).

The new species closely resembles *Donacia bicolora* Zschach, but can be distinguished by the following diagnostic characteristics: the rows of punctures are still clearly visible even in the last third of the elytra (*D. bicolora*: these punctures are almost completely obscured by the silky, shiny, fine wrinkles in the last third of the elytra).; and it generally occurs in two distinct color morphs: cupreous and greenish (*D. bicolora* exhibits a variety of body colorations, commonly greenish, bluish, or blackish). The legs are entirely metallic.

**Figure 1.**
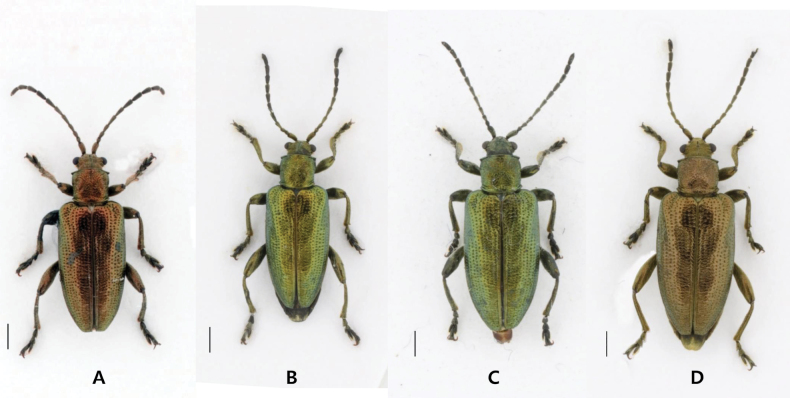
*Donacia koreana* sp. nov. **A, B.** Male (**A.** Holotype); **C, D.** Female (**C.** Paratype). Scale bars: 1.0 mm (**A–D**).

**Figure 2.**
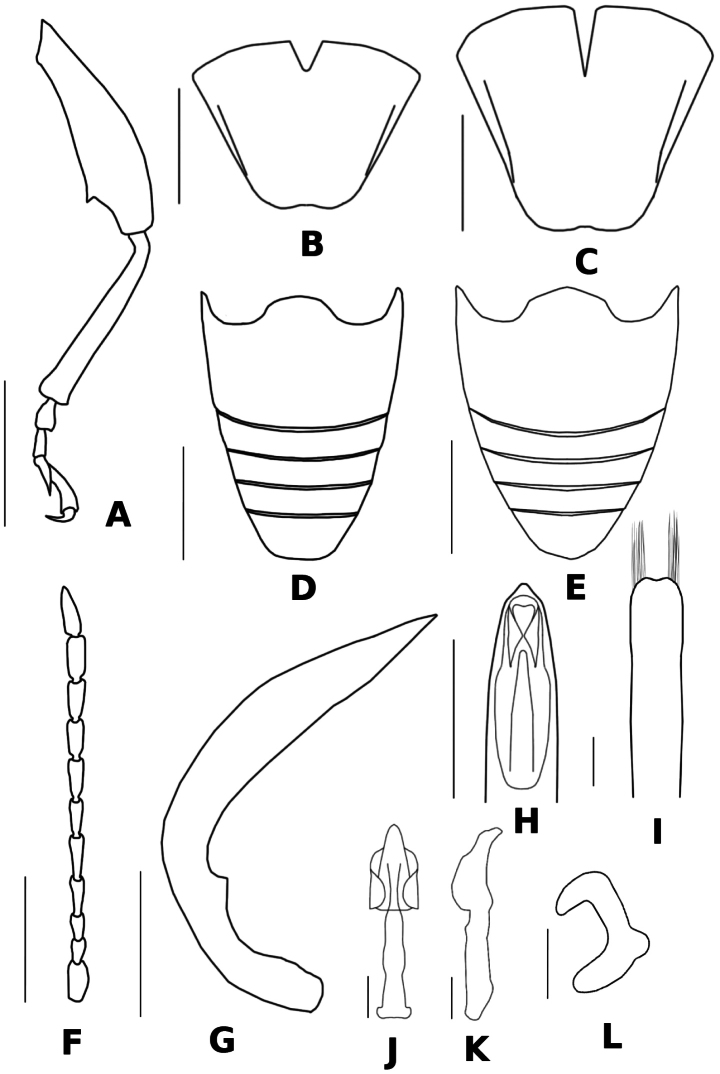
Adult: *Donacia koreana* sp. nov. **A.** Leg (l.v.); **B, C.** Pygidium (d.v.); **D, E.** Abdomen (d.v.); **F.** Antenna (d.v.); **G.** Median lobe (l.v.); **H.** Apex of median lobe (d.v.); **I.** A cap of tegmen; **J, K.** Endophallus (d.v. and v.v.); **L.** Spermatheca. male: **A, B, D, F–K**; female: **C, E, L.** Scale bar: 1.0 mm (**A, D, E, F**); 0.5 mm (**B, C, G, H**); 0.2 mm (**I–L**).

#### Description, adult (Figs 1, 2, 4).

**Male.** Length 7.9–8.1 mm, width 2.7–2.8 mm. Body narrowly elongate; generally cupreous or greenish; head cupreous or greenish; antennomeres 1–4 coppery or greenish, 5 partially darkened, 6–11 dark; pronotum cupreous or greenish; legs cupreous or greenish.

***Head*.** Narrower than prothorax; eyes small and convex; supraocular furrow present but shallow; vertex slightly swollen, pubescent with a fine median line. Antenna filiform and pubescent; antennomere 5 longest amongst 2^nd^ to 6^th^; antennomere 4 slightly longer than 3^rd^; antennomere 5 2.5 times longer than 2^nd^.

***Pronotum*.** Generally quadrate, width as long as length, posterior and anterior corners prominent; anterolateral calli present, callosal sulcus present but shallow; median line deep; disc coarsely punctate and transverse deep rugae; basal sulcus present but shallow, coarsely punctate.

***Scutellum*.** Subtriangular, closely covered with silvery pubescent.

***Elytron*.** Subparallel-sided, gradually narrowed apically; surface distinctly 10 longitudinal regular striate, striae regular and deeply punctate; with a shallow depression at the base near the suture (1^st^–4^th^ sutural intervals), and a deep depression at the base of the median area (5^th^–8^th^ sutural intervals); apex truncate, outer apical angle obtusely rounded, inner apical angle obtuse.

***Legs*.** Slender; metafemur robust in shape, tapering and not distinctly clavate, with a prominent large tooth.

***Pygidium*.** Trapezoidal and apex pubescent, emarginate in both sexes.

***Male genitalia*.** Median lob of genitalia narrowed apically with median lip and both sides emarginate subapically; a cap of tegmen slender, apex with shallow depression; endophallus elongate, median process short and curving dorsal, BSB elongate.

***Sterna*.** Entirely greenish and pubescent; apical shape of last sternite truncate.

**Female.** Length 9.0–9.3 mm, width 2.8–3.1 mm. Body larger than that of males; pronotum frontal outline quadrate, width longer than length; apical shape of last sternite pointed.

***Spermatheca*.** Short and broad, strongly C-shaped; apex tapered; collum and ramus distinct; cornu and nodulus fused into a globose basal swelling; horns widely separated.

#### Description, first instar larva.

Length 1.66–1.78 mm, width 0.40–0.41 mm, spiracle-hook length (0.213 mm), and spiracle-hook width (0.033 mm (*n* = 4). Creamy white, body slightly convex, thorax and abdomen with numerous setae, but not as setae as the last instar larva; egg buster absent.

#### Diagnosis, last instar larva (Figs 3, 4C).

The last instar larva is easily distinguished from the other *Donacia* larvae by the following characteristics: epicranium with six pairs of dorsal setae; two pairs of sensilla; labrum with three pairs of labral setae and one pair of sensilla; and prementum with one pair of setae and two pairs of sensilla.

**Figure 3.**
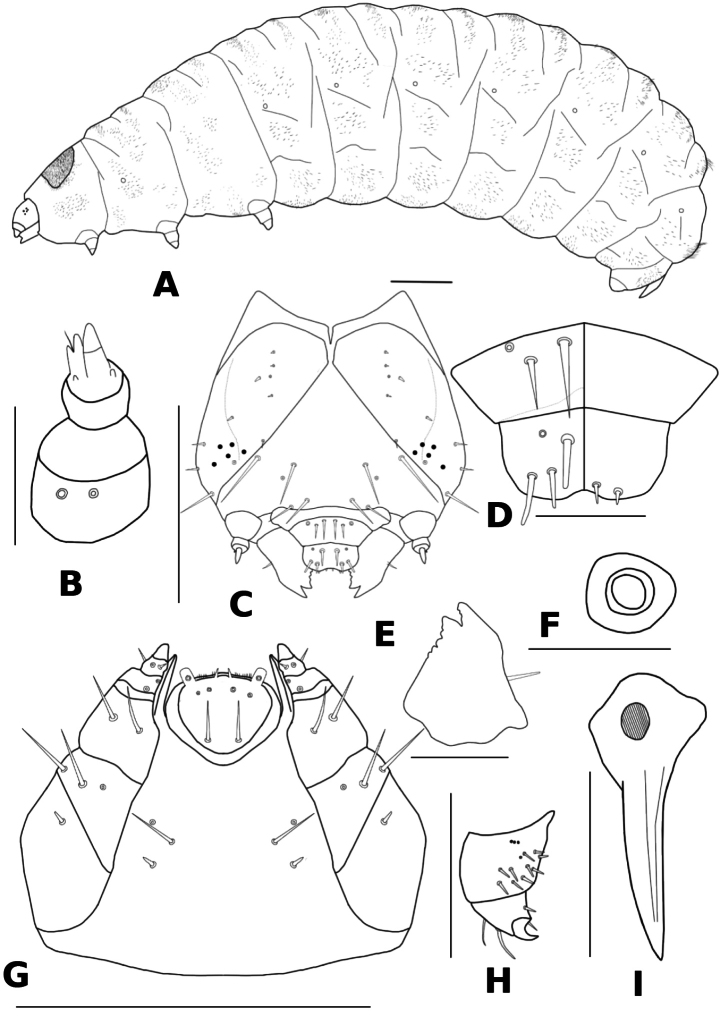
Larva: *Donacia koreana* sp. nov. **A.** Last instar larva (l.v.); **B.** Antenna (d.v.); **C.** Head (d.v.); **D.** Clypeus, labrum and epipharynx (d.v. and v.v.); **E.** Mandible (b.v.); **F.** Spiracle (d.v.); **G.** Maxilla and labium; **H.** Leg (v.v.); **I.** 8^th^ abdominal spiracle. Scale bar: 1.0 mm (**A**); 0.5 mm (**C, H, G, I**); 0.1 mm (**B, E, F**).

**Figure 4.**
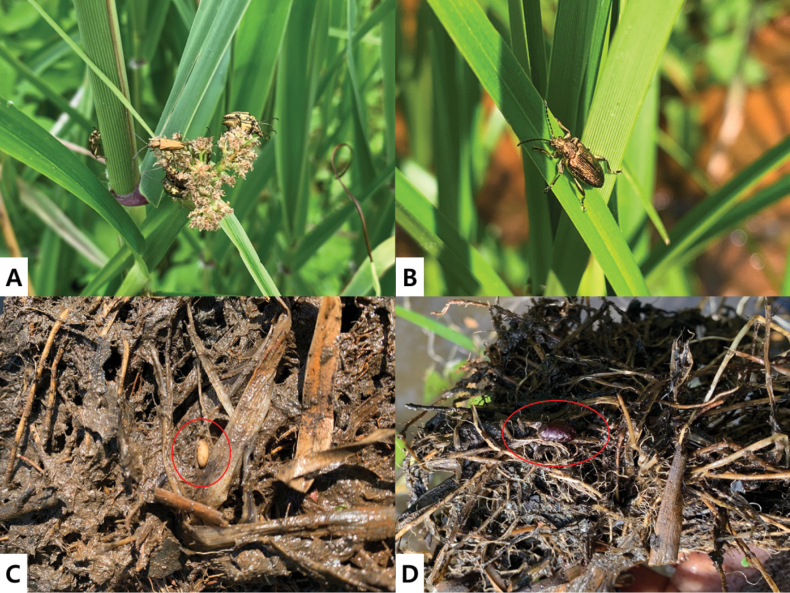
*Donacia koreana* sp. nov. **A, B.** Adults; **C.** Larva; **D.** Pupal case; (**C, D** red circle).

#### Description, last instar larva (Figs 3, 4C).

Length 10.5–12.3 mm, width 3.7–4.3 mm, head length 0.78–0.84 mm, spiracle hook length 0.73–0.77 mm, spiracle hook width 0.42–0.44 mm (*n* = 5). Body yellowish, slightly convex, and C-shaped. Head, prothorax, and legs pale brown; setae and spiracles brown.

***Head*.** Prognathous, small, rounded, well sclerotized, strongly retractile into prothorax; hind corner of epicranium slightly posteriorly produced. Epicranial suture Y-shaped; frontal suture nearly straight. Stemmata pigmented, five in number. Epicranium with six pairs of dorsal setae, two pairs of sensilla, and three pairs of lateral setae. Frons with four pairs of frontal setae and one pair of sensilla. Antenna three-segmented: segment 1 with two sensilla, segment 2 with conical sensory papilla and two sensilla basiconica, and segment 3 with two sensilla basiconica and one seta. Endocarina absent; epistomal sutures distinct. Clypeus with two pairs of clypeal setae and one pair of sensilla. Labrum slightly notched anterior margin with three pairs of labral setae and one pair of sensilla. Epipharynx with two pairs of spiniform setae. Mandible conical, strongly sclerotized, with two teeth, 2^nd^ serrated, and one mandibular seta. Maxillar palp three-segmented; palpifer with two setae; stipes, two setae and one sensillum; galea fused with lacinia, spear-like; cardo large, trapeziform, with one seta. Labial palp one-segmented with one sensillum; ligula with numerous setae; prementum with one pair of setae and two pairs of sensilla; postmentum with two pairs of setae and one pair of sensilla.

***Thorax*.** Pronotum pale brown, slightly sclerotized, with numerous setae. Meso-and metanotas not sclerotized. Thoracic spiracles annuliform, situated on the EPa; spiracular opening rounded. Legs short and stout; femur with ten setae and four sensilla; tibio-tarsus with three setae; claws slightly curved, awl-shaped, based enlarged, with one seta; pulvillus absent.

***Abdomen*.** Nine segmented, unsclerotized. Typical abdominal segments with two folds. Abdominal spiracles present on segments 1–8 similar to mesothoracic spiracles except for 8^th^ spiracle. Spiracle hook length about 2.3 times as long as width; projected, large, peg-like, strongly sclerotized.

#### Distribution.

South Korea.

#### Host plants.

Cyperaceae: *Scirpus wichurae* Boeckeler, *Eleocharis ussuriensis* G. Zinserl.

##### ﻿Key to the adults of South Korean *Donacia* species

**Table:** 

1	Entire dorsum of the beetle not really metallic at all; appendages predominantly bicolored to rufescent; pronotal disc with at most fine punctures and rugulae; elytra with at most few fine rugulae and minute punctulae in intervals	**2**
–	Entire dorsum of beetle metallic; appendages predominantly dark; pronotal disc coarsely punctured and often transversely coarsely rugose; elytral disc generally coarsely rugose between striae	**3**
2	Third antennal segment slightly longer than, or as long as second	***D. lenzi* Schönfeldt**
–	Third antennal segment much longer than second	***D. provostii* Fairmaire**
3	Femora and tibia entirely metallic-colored	**4**
–	Femora and tibia partly metallic-colored (femoral base usually rufous)	**5**
4	Elytron with red longitudinal stripe	***D. aquatica* (Linnaeus)**
–	Elytron without red longitudinal stripe	**6**
5	Pronotal disc with distinct fine tum with short hairs (most specimens)	***D. clavareaui* Jacobson**
–	Pronotal disc without distinct short hairs	***D. bicoloricornis* Chen**
6	Body and legs entirely black, unicolored	***D. flemola* Goecke**
–	Body and legs entirely cupreous or green	***D. koreana* sp. nov.**

##### ﻿Key to the larvae of South Korean *Donacia* species

**Table:** 

1	Head inside the prothorax	**2**
–	Head outside the prothorax	***D. flemola* Goecke**
2	Hind corners of epicranium moderately produced	**3**
–	Hind corners of epicranium slightly produced	**4**
3	Ephipharynx with three pairs of spiniform setae	***D. clavareaui* Jacobson**
–	Epipharynx with two pairs of spiniform setae	***D. koreana* sp. nov.**
4	Head ovoid	**5**
–	Head rounded	**6**
5	Postmentum with two pairs of setae	***D. lenzi* Schönfeldt**
–	Postmentum with three pairs of setae	***D. aquatica* (Linnaeus)**
6	Clypeus with two pairs of clypeal setae	***D. bicoloricornis* Chen**
–	Clypeus with three pairs of clypeal setae	***D. provostii* Fairmaire**

###### ﻿Biological note

##### ﻿Feeding pattern

To investigate the feeding behavior of the Korean Donaciinae, the classification system proposed by Bieńkowski (2015) was adopted. According to this framework, three distinct feeding patterns were identified.

- Type I: where the insect inclines its head, turns it to the right or left, and inserts both mandibles into the leaf tissues.
- Type II: characterized by the insertion of one mandible into the leaf, whereas the other remains on the leaf surface.
- Type III: the head movement of the insect resembles that of Type II, but both mandibles capture the upper and lower surfaces of the leaf.

**Figure 5.**
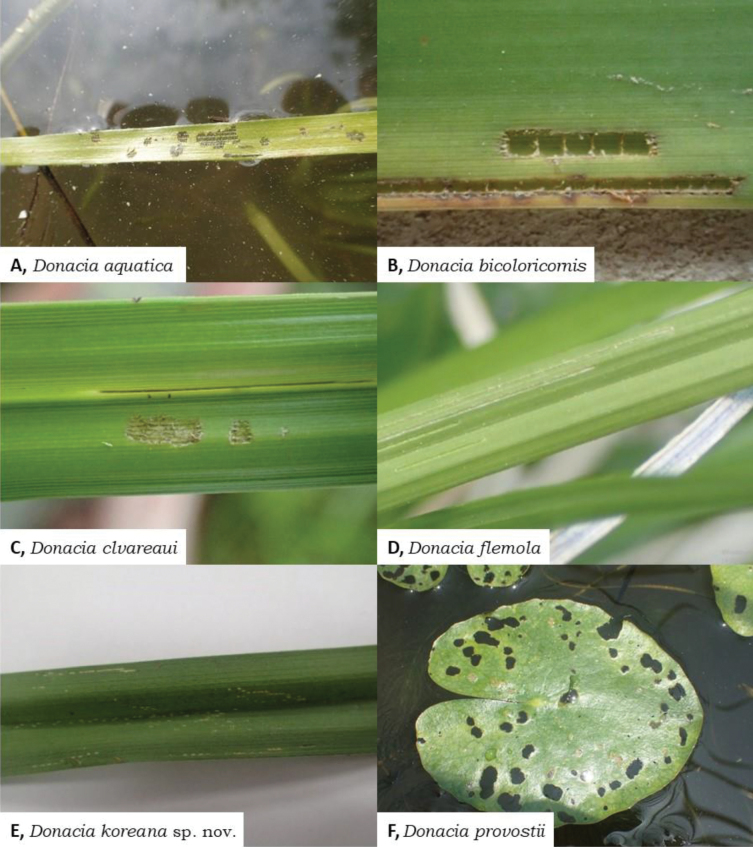
Feeding pattern of Korean *Donacia*. **A, B.** Type I; **C, D.** Type II; **E, F.** Type III.

Based on the observations following this classification, *D. lenzi* and *D. provostii* exhibited Type I feeding behavior. *Donacia aquatica*, *D. bicoloricornis*, and *D. clavareaui* displayed Type II feeding behavior, whereas *D. flemola* and *D. koreana* sp. nov. exhibited Type III behavior (Fig. 5). Notably, although *D. koreana* sp. nov. engaged in leaf feeding, its primary feeding sites were the flowers of *Scirpus wichurae* and *Eleocharis ussuriensis* rather than leaf tissues.

##### ﻿Oviposition patterns

Adult Donaciinae exhibit diverse oviposition behaviors that vary depending on the species and associated host plant (Bieńkowski 1996). In the present study, oviposition types were identified in six species using a combination of laboratory rearing and field observations. Based on these findings, oviposition behaviors can be categorized into three distinct types.

- Type I: eggs are deposited between overlapping leaves floating on the water surface and subsequently covered with a protective membrane.
- Type II: eggs are laid inside the stem near the water surface and enclosed by a membrane.
- Type III: eggs are deposited along the feeding trace on the abaxial side of the host plant and covered with a membrane.

**Figure 6.**
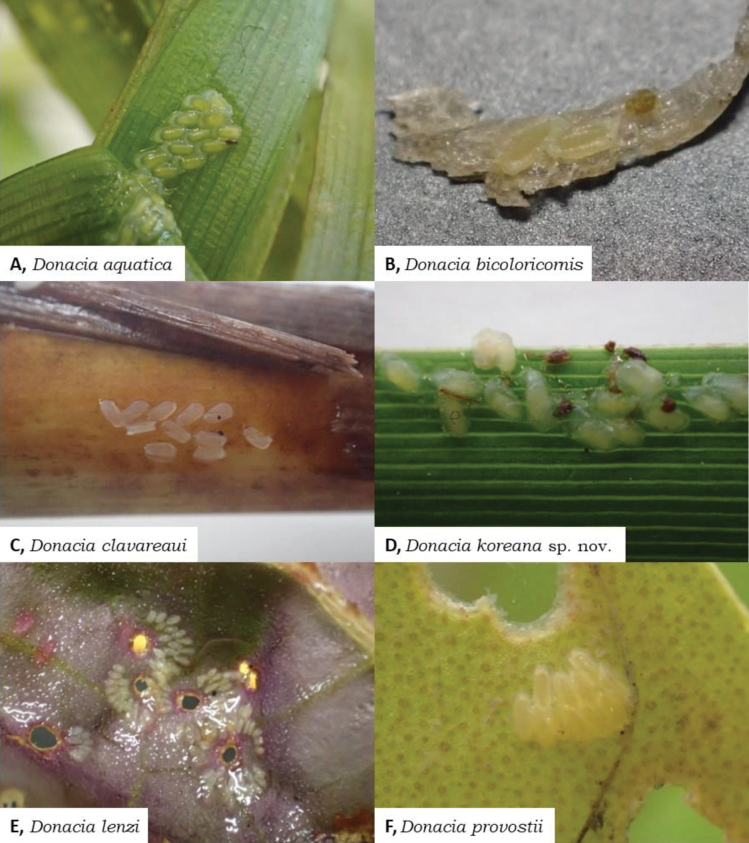
Oviposition patterns of Korean *Donacia*. **A, B.** Pattern I; **C, D.** Pattern II; **E, F.** Pattern III.

Based on these classifications, *D. aquatica* and *D. koreana* sp. nov. exhibited Type I oviposition behavior. *Donacia bicoloricornis* and *D. clavareaui* exhibited type II oviposition, whereas *D. provostii* and *D. lenzi* exhibited Type III oviposition (Fig. 6).

##### ﻿Habitat and distribution

**Figure 7.**
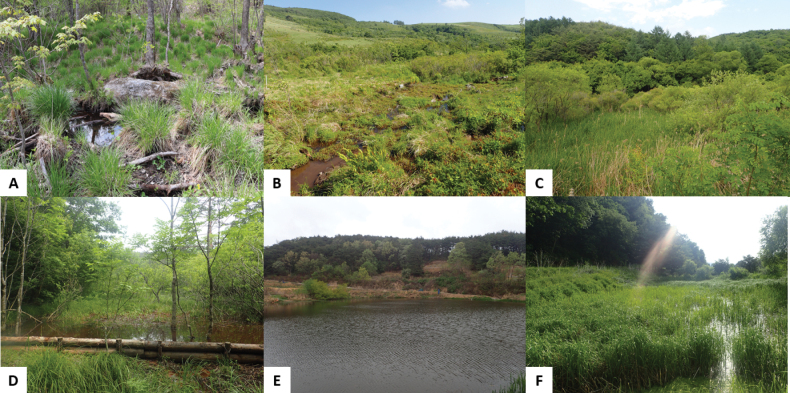
Habitat of genus *Donacia*. **A, B.** High-elevation wetland; **C, D.** Mid-elevation wetland; **E, F.** Low-elevation wetland.

**Figure 8.**
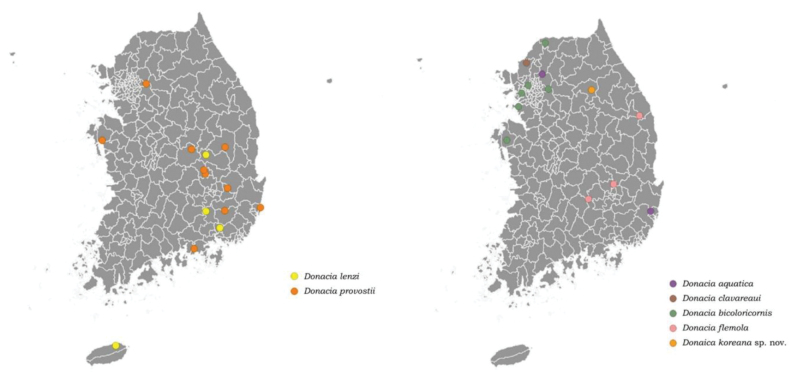
Distribution map of genus *Donacia*.

The primary habitats of the Korean *Donacia* species are aquatic environments with low water flow such as reservoirs, ponds, paddies, rivers, and waterside areas with gentle currents. These habitats are classified as low-, mid-, or high-elevation wetlands based on their altitude. Each species exhibits slight differences in habitat preferences, including environmental conditions, wetland type, and elevation. *Donacia lenzi* and *D. provostii* primarily inhabit lowland riverbank wetlands, reservoirs, and slow-flowing streams. *D. aquatica* and *D. bicoloricornis* are primarily found in reservoirs and low-elevation wetlands. *D. clavareaui* occurs in lowland wetlands, *D. flemola* is found in high-elevation wetlands, and *D. koreana* sp. nov. primarily inhabits mid-elevation wetlands (Table 2, Figs 7, 8).

**Table 2.** Habitat of Korean *Donacia*.

Species	Habitat	Altitude (average)
*Donacia aquatica*	paddy, pond, reservoir, low-elevation wetland	102–111 m (106 m)
*Donacia bicoloricornis*	reservoir, pond	3–189 m (45 m)
*Donacia clavareaui*	low-elevation wetland	12–102 m (57 m)
*Donacia flemola*	high-elevation wetland	539–932 m (815 m)
*Donacia koreana* sp. nov.	middle-elevation wetland	539 m
*Donacia lenzi*	reservoir, low-elevation wetland	1–109 m (40 m)
*Donacia provostii*	river, reservoir, low-elevation wetland	1–94 m (54 m)

According to the aquatic plant habitat classification proposed by van der Valk (2012), aquatic plants are divided into four categories based on their growth forms: terrestrial wetland plants, amphibious plants, floating-leaved aquatic plants, and submerged aquatic plants.

**Figure 9.**
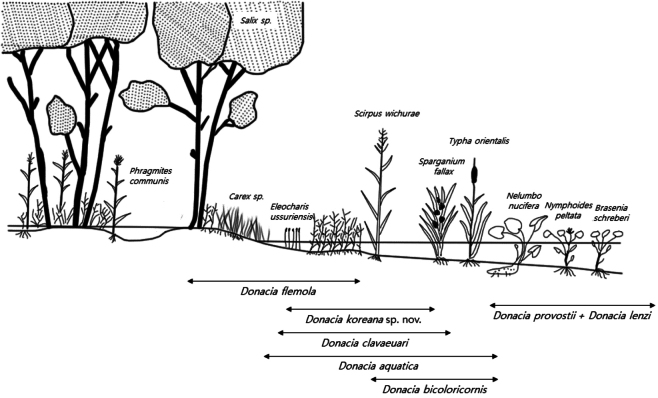
Habitat of each species of Korean *Donacia* (modified after Fossil Insect Research Group for Nojiri-ko Excavation 1985).

The habitats of the Korean *Donacia* species can be classified as follows:

*D. lenzi* and *D. provostii* prefer habitats dominated by submerged aquatic plants, with floating leaves (Nymphaeaceae). By contrast, *D. aquatica*, *D. bicoloricornis*, *D. clavareaui*, *D. flemola* and *D. koreana* sp. nov. prefer habitats with terrestrial wetland plants (Fig. 9).

##### ﻿Molecular data

**Figure 10.**
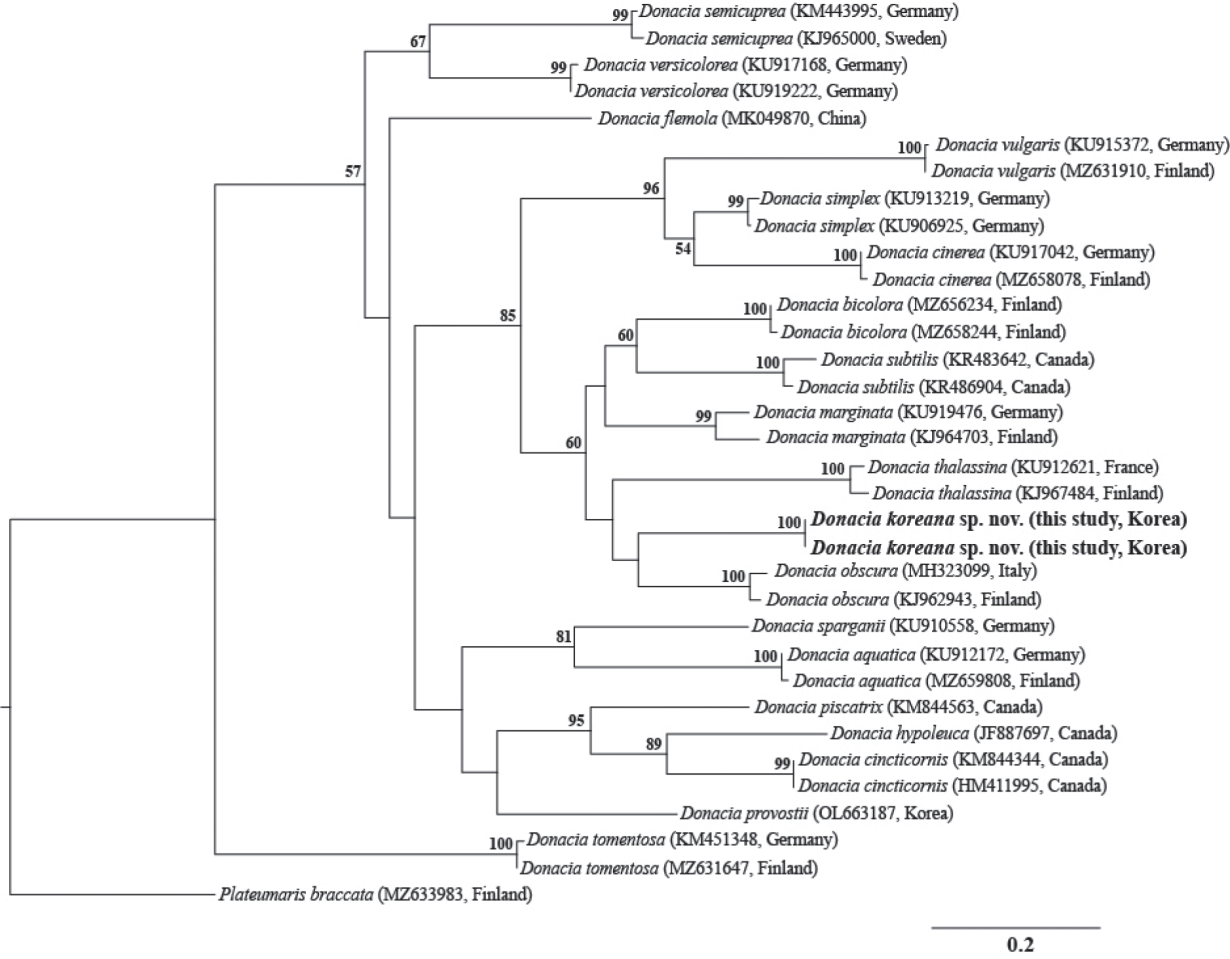
Maximum-likelihood phylogenetic tree based on *COI* gene sequences. Bootstrap support values (>50%) are indicated above the branches. *Plateumaris braccata* was used as an outgroup taxon.

We obtained a 658-bp fragment sequence of the *COI* gene from two individuals of *D. koreana* sp. nov., and the *COI* sequences were deposited in GenBank under accession numbers PX400636 and PX400637. Genetic distance analysis based on a 564 bp fragment of the *COI* gene showed that intraspecific divergence among *Donacia* species ranged from 0.0% to 3.9%, with *D. koreana* sp. nov. exhibiting no genetic variation (0.0%). Interspecific genetic distances within the genus *Donacia* ranged from 9.2% to 22.9%, whereas those between *D. koreana* sp. nov. and *D. obscura* ranged from 11.2% to 11.4% (Fig. 10).

## Supplementary Material

XML Treatment for Donacia koreana

## ﻿Additional information

### Use of AI

No use of AI was reported.

### Author ORCIDs

Joong Youb Kim https://orcid.org/0009-0008-2271-3104

Yeong-Deok Han https://orcid.org/0000-0002-6625-8966

Jong Eun Lee https://orcid.org/0000-0003-0536-0032

### Data availability

All of the data that support the findings of this study are available in the main text.
